# Risk factors for failing endotracheal extubation in neurocritical patients: a retrospective cohort study

**DOI:** 10.3389/fneur.2025.1562454

**Published:** 2025-03-26

**Authors:** Xintong Zhang, Shuang Zheng, Chen Chen, Sifan Wang, Yajuan Hu

**Affiliations:** ^1^Department of Neurology, Neurocritical Care Unit, The First Affiliated Hospital of Anhui Medical University, Hefei, Anhui, China; ^2^The First Affiliated Hospital of USTC, Division of Life Sciences and Medicine, University of Science and Technology of China, Hefei, Anhui, China; ^3^Collaborative Innovation Center of Neuropsychiatric Disorders and Mental Health, Hefei, China; ^4^The School of Mental Health and Psychological Sciences, Anhui Medical University, Hefei, Anhui, China

**Keywords:** neurocritical care, endotracheal extubation, airway control, Glasgow Coma Scale, risk factors

## Abstract

**Objective:**

To identify risk factors of failing endotracheal extubation among neurocritical care patients with endotracheal intubation for more than 48 h and passing the autonomous breathing test (SBT) and establish a prediction model accordingly.

**Methods:**

This study included the clinical data of patients who received standardized monitoring and treatment in the neurocritical care unit of the First Affiliated Hospital of Anhui Medical University from April 2020 to August 2024. Based on the outcomes of extubation after 5 days, data were divided into the success group and the failure group. Clinical features of two groups were compared and accordingly taken into multivariate logistic regression analysis, eventually generating a scoring model with its receiver operating characteristic curve (ROC). The area under the curves (AUC) of other previous scores was compared by Z-test.

**Results:**

Of 116 recorded cases, 92 (79.3%) were successfully extubated, while 24 (20.7%) required re-intubation within 5 days. Univariate analysis revealed significant differences between two groups in state of consciousness, Glasgow Coma Scale (GCS) total score, GCS motor score (GCS-M), muscle strength, swallowing ability, coughing response, body temperature, oxygenation index, Apache II score, and APS score (all *p* < 0.05). Multivariate analysis was further carried out, and a scoring model was established accordingly (including GCS-M, coughing ability, and oxygenation index) with a total score of 4 points. The model demonstrated good predictive value, with a cut-off ≥1 distinguishing extubation success with 79.2% sensitivity and 69.6% specificity according to ROC (AUC = 0.79; 95% CI, 0.68–0.90).

**Conclusion:**

This clinical predictive scoring model could provide guidance for extubation decisions in neurocritical care units but requires further external validation.

## 1 Introduction

Failed extubation, generally defined as the need for re-intubation or tracheotomy within 48 h of planned extubation, can lead to Mechanical injury and hypoxia, prolonged hospitalization, worse prognosis, and even increased mortality. Therefore, the assessment of risk factors for failed extubation and strategies for optimizing extubation success are essential for the clinical management of intensive care unit (ICU) patients (1). Passing the spontaneous breathing trial (SBT) is an important prerequisite for extubation (2). Conversely, unnecessary delays in extubation can also result in deleterious outcomes such as increased risk of ventilator-associated pneumonia, prolonged hospitalization, and higher mortality even among patients passing the SBT and successfully extricated from the ventilator (3). The overall extubation failure rate following SBT is reported to be about 10–20% (4), but can be as high as 38% in brain injury patients (5), so tracheotomy is frequently required within 48 h for many intubated neurocritical patients. This decision is often based on the “Stroke-Related Early Tracheotomy (SET) Score” (6). However, a recent multicenter study found that patients who underwent early tracheostomy according to the SET score did not have a better prognosis at 6 months than those who had to undergo tracheostomy after 10 days of intubation (7). Moreover, earlier tracheotomy deprived some patients of the benefits of direct extubation. Therefore, a major current concern in critical care medicine is how best to predict which neurocritical patients will benefit from extubation at a given time.

Previous studies have identified coma, poor airway protection, severe lung infection, advanced age, and combined underlying chronic cardiopulmonary diseases as predictive factors for unsuccessful extubation (8), but most of these studies enrolled comprehensive ICU patients. Alternatively, the primary pathologies of neurocritical patients, such as traumatic brain injury (TBI), cerebrovascular disease, encephalitis, hypoxic–ischemic encephalopathy, and central demyelinating disorders, and the secondary sequela such as decreased level of consciousness, poor airway protection, and potential damage to the respiratory drive or respiratory motor conduction pathway, may differentially influence extubation failure risk among this clinical group. In addition, the rates of extubation failure, delayed extubation, and tracheotomy are generally higher among neurocritical patients even if respiratory parameters are satisfactory during mechanical ventilation (9). Therefore, the risk factors for extubation failure among neurocritical patients may be distinct and potentially missed in studies of the general ICU population.

For these reasons, the investigation of risk factors for extubation failure requires neurological assessment in addition to evaluation of respiratory parameters (10). Godet and colleagues reported that lower Glasgow Coma Score (GCS) is associated with greater risk of extubation failure (11), but McCredie and associates reported that some patients with impaired consciousness can be successfully extubated, and that age, choking reflex function, and fluid balance are more strongly predictive of extubation success (12). There are several clinically relevant scores available for predicting the risk of failed extubation, including the simplified Clinical Utility Score developed by Godet and colleagues based on assessment of coughing, swallowing, gag reflexes, and neurologic status (11), the VISAGE score is based on visual pursuit, swallowing, age, and GCS (13), the Respiratory Insufficiency Syndrome Intubation Scale-intubated (RIS-i) score based on level of sedation, oxygenation, cough reflex, and swallowing (14), and the Extubation Strategies in Neuro-Intensive Care Unit Patients and Associations With Outcome (ENIO) scale (9). However, most previous studies have used 48 h after extubation as the cut-off for distinguishing success from failure. Moreover, patient group sizes were relatively small with the exception of the ENIO score study.

In clinical practice, many neurocritical patients require re-intubation several days after extubation, and these patients should be counted as extubation failures. Therefore, set 5 days after extubation as the observation cut-off and found that this longer duration encompassed >90% of total failure cases (15). In the present study, we compared demographic characteristics, neurological status indices, airway protection capacity, circulatory function, and respiratory function between extubation success and failure subgroups of neurocritical patients to develop a predictive scoring scheme for extubation. Further, we provide external validation of several other scoring systems.

## 2 Materials and methods

### 2.1 Study population and setting

**Figure 1:**
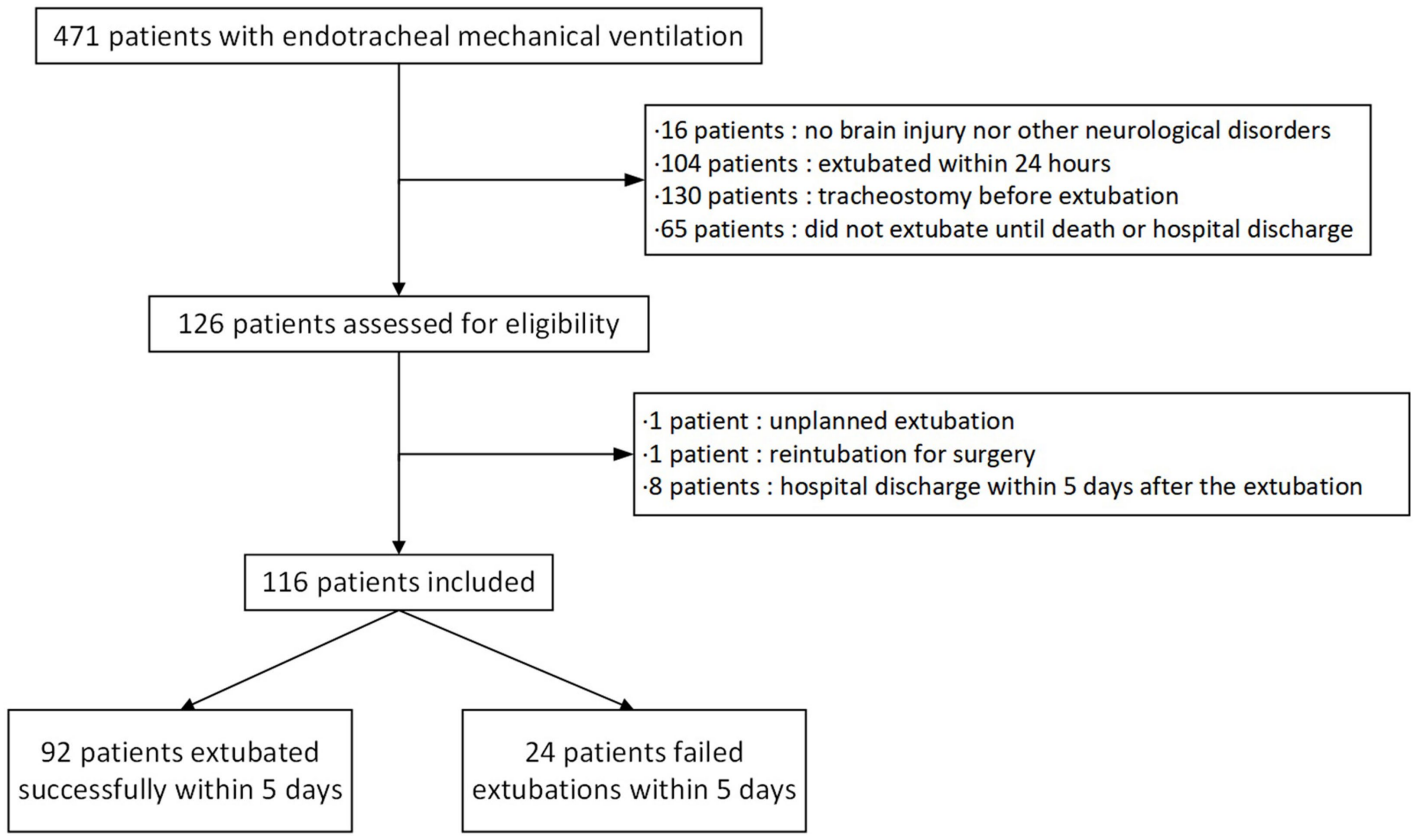
Flowchart of data collection.

The study was conducted at the ICU of the Department of Neurology (Neurocritical Intensive Care Unit, NICU), the First Affiliated Hospital of Anhui Medical University. Neurocritical patients ≥18 years old requiring endotracheal intubation for over 24 h were screened as study candidates between April 2020 and August 2024 (Figure 1).

Inclusion criteria were as follows: (1) patients diagnosed with TBI, subarachnoid aneurysmal hemorrhage (SAH), intracranial hemorrhage (ICH), ischemic stroke, hypoxic–ischemic encephalopathy (HIE), metabolic encephalopathy, central nervous system infection, encephalitis, or brain tumor; (2) receiving endotracheal intubation for more than 24 h; (3) successfully passing the SBT without any significant deterioration in mental status (16). The SBT requirements were maintenance on intubated oxygen for >2 h with stable blood pressure (systolic <180 mm Hg or > 90 mm Hg), stable heart rate (<120/min or variance <20%), and stable respiration (RR < 35/min, SpO^2^ > 90%). Exclusion criteria included (1) unplanned extubation or autoextubation, (2) tracheotomy, (3) withdrawal of care or automatic discharge within 5 days after extubation.

This study meets the standards of medical ethics and is approved by ethics committee of the First Affiliated Hospital of Anhui Medical University (PJ20231136).

### 2.2 Extubation protocol

After the patient passed the SBT, the physician in charge assessed the level and content of consciousness, airway protection ability, and choking risk. Airway protection ability was evaluated by observing the degree of oral salivary accumulation and the presence of spontaneous thyroid cartilage displacement indicative of swallowing. Further, the risk of choking was assessed by monitoring the frequency and intensity of cough produced during sputum suctioning and the amount, thickness, and blood content of airway secretions.

Once extubation is decided, 5 mg of dexamethasone was administered routinely through intravenous injection half an hour in advance, then the nurse suctioned the oral cavity and nasal catheter secretions, and provided inhaled oxygen via the nasal catheter for 2–3 min. The gas in the air sac was emptied while maintaining negative pressure of the sputum suction tube, and the tracheal tube pulled out. The patient then received aerosol inhalation, ordinary nasal catheter oxygen inhalation, or hyperthermia and humidification high-flow oxygen therapy according to lung function. Some patients were irritable due to primary disease and intubation discomfort, so low-dose sedatives were administered before extubation (dextromethorphan about 0.3 mg/kg/h or midazolam about 0.5 mg/kg/min) to maintain a Ramsay score of 2 or 3 points (17).

### 2.3 Data collection

At a practical level, documentations as much detail as possible of demographic and clinical data was collected by two experienced neurologists and a nurse through the hospital’s electronic record system and actual clinical observations. It is difficult to assess language function during tracheal intubation, so the verbal score of the GCS was set to 1 point (18). When assessing limb movements in people with impaired consciousness, stimulation is performed by pressing on the patient’s supraorbital foramen, and muscle strength grading was based on the highest muscle strength of the upper limb on the left or right side. Airway protection ability was classified from strong to weak according to the following criteria: Grade I, strong swallowing action with no saliva accumulation; II, weak swallowing action with a small amount of saliva outflow; III, no swallowing action and a large amount of saliva outflow. Coughing ability was similarly categorized as follows: I, strong coughing, more than 4 consecutive sounds, and sputum choking out of the catheter; II, medium coughing, 2–4 sounds, and sputum not choked out of the catheter; III, weak coughing, less than 2 sounds; IV, no response to sputum suction. Physiological parameters recorded included vital signs, oxygenation index, arterial blood gas concentrations, hemoglobin content, hematocrit, platelet count, white blood cell count, percentage of neutrophils, liver and kidney function variables, and electrolyte concentrations. We also collected the data required to calculate Godet’s, VISAGE and RIS-i scores according to the methods detailed here and in previous studies. For original RIS-i score calculation, mental status is assessed using the Richmond Agitation Sedation Score (RASS), the scale is divided into 10 levels of sedation, from +4 points to −5 points represent the degree of the patient from “aggressive” to “coma,” and each score corresponds to a state of consciousness. However, it is not usually used for neurological consciousness assessment. Therefore, we replaced the RASS score for RIS-i score calculation with a simple grade of consciousness as follows: awake = 3, drowsiness = 2, lethargy = 1, coma = 0. We then screened the potential risk factors of failing extubation for further analysis from both the perspective of the diseases’ pathophysiology included in this study and other previous studies mentioned in Introduction.

### 2.4 Statistical analysis

All statistical analyses were conducted using IBM SPSS Statistics 27.0. Dichotomous variables were compared by chi-square test or Fisher’s exact test as indicated; independent samples t-test is used when the continuous variable conforms to a normal distribution. The remaining variables including the hierarchical data and skewed distribution data are tested by the Mann–Whitney U-test. Based on the results of this univariate analysis (*p* < 0.05) and clinical correlation, multivariate analysis was performed using a logistic regression model, and the results expressed as the odds ratio (OR) and 95% confidence interval (95%CI). According to the logistic regression results, the scoring system of this study was established. The corresponding receiver operating curve (ROC) and area under the curve (AUC) were obtained as well. Then the optimal cut-off value was determined by maximizing the Youden index (Youden’s J = sensitivity + specificity −1). Z-test was used to compare the AUCs of other previous models mentions in Introduction. A post-hoc power analysis was conducted using G*Power 3.1.

## 3 Results

### 3.1 Patient characteristics

A total of 116 patients (Figure 1) were included in the statistical analysis (ranged 18–92 years, 72 males [62.0%]). The most frequent etiology was stroke (*n* = 81, 69.83%), followed by traumatic brain injury (*n* = 14, 12.07%), central nerve system infection and secondary epilepsy (*n* = 9, 7.76%), spinal cord injury (*n* = 2, 1.72%), Parkinson’s disease (*n* = 2, 1.72%),acute and chronic inflammatory demyelinating polyradiculoneuropathy (*n* = 2, 1.72%), myasthenia gravis (*n* = 2, 1.72%), ischemic–hypoxic encephalopathy (*n* = 1, 0.86%), brain tumor (*n* = 1, 0.86%) and disorders of consciousness due to other causes (*n* = 2, 1.72%). The duration of intubation ranged from 2 to 18 days. 92 patients in this cohort were successfully extubated while 24 patients were not (failure rate of 20.69%). Median duration of intubation did not differ significantly between the extubation success and failure groups (112 h vs. 90 h, *p* = 0.23). Of these failures, 12 (50%) occurred <24 h after extubation, 4 (16.67%) between 24 and 48 h, 4 (16.67%) between 48 and 72 h, and 4 (16.67%) between 72 and 120 h. The reasons for re-intubation in the failure group were mainly weak cough and unmanageable endotracheal secretion (*n* = 14, 58.33%) and hypoxemia (*n* = 10, 41.67%). Airway narrowing due to edema or spasticity of the airway was found on re-intubation in 4 patients (16.67%), while glossoptosis was found in 3 patients (12.5%), cardiovascular failure in 1 (4.17%), and respiratory muscle weakness in one (4.17%). After extubation failure and re-intubation, 20 patients required tracheostomy (83.33%), while 4 (16.67%) were successfully re-extubated again. Based on the information collected, we additionally calculated acute physiology and chronic health evaluation (Apache II) with its acute physiology score (APS) component, and sequential organ failure assessment (SOFA) score ahead of extubation to represent the patient’s overall physiology at that time (19, 20) (Table 1).

**Table 1:** Univariate analysis between extubation success group and failure group.

	Extubation success (*n* = 92)	Extubation failure (*n* = 24)	*χ^2^/t/Z*	*p*
Age, median (IQR)	63 (51.25–73.0)	67 (57.25–74.75)	−1.37	0.17
Sex (male), n (%)	59 (64.1%)	13 (54.2%)	0.80	0.37
Stroke, n (%)	63 (68.4%)	18 (75.0%)	0.38	0.54
Endotracheal intubation time, hour, median (IQR)	112 (72, 192)	90 (48, 162)	−1.21	0.23
GCS Total Score, median (IQR)	10 (8.25, 11)	7.25 (5.25, 11)	−2.60	0.009
GCS(E), median (IQR)	4 (3, 4)	3 (3, 4)	−0.93	0.36
GCS(M), median (IQR)	6 (5, 6)	4 (1, 6)	−3.58	<0.001
Muscle strength (0–5), median (IQR)	4 (3, 5)	2 (0, 3)	−4.22	<0.001
Level of consciousness, n (%)			−2.54	0.011
Awareness	36 (39.1%)	6 (25%)		
Sleepiness	41 (44.6%)	7 (29.2%)		
Lethargy	5 (5.4%)	1 (4.2%)		
Coma	10 (10.9%)	10 (41.7%)		
Cough, n (%)			−2.11	0.034
I	30 (32.6%)	5 (20.8%)		
II	48 (52.2%)	13 (54.2%)		
III	12 (13.0%)	5 (20.8%)		
IV	2 (2.2%)	1 (4.2%)		
Swallow, n (%)			−1.38	0.018
I	50 (54.3%)	6 (25%)		
II	39 (42.4%)	15 (62.5%)		
III	3 (3.3%)	3 (12.5%)		
Temperature, n (%)			4.418	0.040
<37°C	45 (48.9%)	6 (25%)		
≥37°C	47 (51.1%)	18 (75%)		
Oxygen inhalation after extubation, n (%)			7.80	0.10
Ordinary Nasal Catheter	17 (18.5%)	3 (12.5%)		
Ordinary Mask	22 (23.9%)	2 (8.33%)		
High Flow Inhaled Oxygen	52 (56.5%)	18 (75%)		
Non-invasive ventilator	0	1 (4.17%)		
No oxygen support	1 (1.1%)	0		
c, PaO_2_/FiO_2,_ median (IQR)	266.5 (213.8, 341.9)	239.68 (187.0, 275.3)	−2.06	0.039
Apache II, median (IQR)	8 (10, 13)	13 (10.5, 16.75)	−2.82	0.005
APS, median (IQR)	5 (7, 9)	9 (6.25, 12)	−2.20	0.028
SOFA, median (IQR)	5 (3, 6)	5 (4, 6.75)	−1.48	0.14
Thomas Godet’s score, median (IQR)	14 (14, 14)	13 (12, 14)	−3.86	<0.001
VISAGE score, median (IQR)	2 (2, 3)	2 (1, 2.75)	−2.77	0.006
RIS-i score, median (IQR)	3 (2, 4.75)	5 (4, 6)	−3.52	<0.001
ENIO-score, median (IQR)	59 (46.8, 70.5)	61.0 (59.0, 86.0)	1.905	0.064

IQR, Interquartile Range.

### 3.2 Univariate analysis of influencing factors for extubation success

Before extubation, GCS (*p* = 0.009), GCS motor response (GCS-M) (*p* < 0.001), muscle strength grade (*p* < 0.001) and level of consciousness (*p* = 0.011) differed significantly between success and failure groups. Uni-variate analysis also proved statistically significant differences in patients’ coughing (*p* = 0.0034), swallowing (*p* = 0.018), body temperature (*p* = 0.040), oxygenation index (*p* = 0.039), Apache II score (*p* = 0.005) and APS score (*p* = 0.028). Further, Godet’s, VISAGE, and RIS-i scores differed significantly between success and failure groups. While ENIO score had no significant difference (*p* = 0.064) (Table 1).

### 3.3 Multivariate analysis of factors independently influencing extubation outcomes

In order to make the model more concise and improve its clinical feasibility, statistically significant factors based on the results above were independently classified: pre-extubation GCS-M (≥ 4, 0; <4, 1), coughing ability (≤II, 0; >II,1), body temperature (<37°C, 0; ≥ 37°C, 1) and oxygenation index (>200, 0; ≤200,1). These four factors were subsequently included in binary logistic regression analysis to find independent influencing factors for extubation outcomes.

It should be noted that swallowing ability was omitted from the analysis for two principal reasons: first, oral intubation may interfere with the assessment of saliva accumulation; second, coughing ability serves as an indicator of bulbar muscle functional recovery, like swallowing does. Concurrently, consciousness level was excluded due to the use of sedatives in a subset of patients. Apache II score and APS score were excluded because of their nature as composite scores and containing many sub-items.

The corresponding regression coefficient *β* was calculated according to the OR of three independent risk factors proved by logistic regression (Table 2), and the factors were assigned scores according to the β (ln(OR)). Then a neurocritical extubation scoring model of this study was established with a summit score of 4 (Table 3). According to the ROC of the model, a score ≥ 1 predicted successful extubation (low risk of failure) with 79.2% sensitivity and 69.6% specificity. At this optimal cut-off, the AUC was 0.79 (95% CI: 0.68–0.90, *p* < 0.001). Further, these same analyses also indicated that the predictive efficacy of the current model exceeded that of the Godet’s score (AUC = 0.68; 95%CI: 0.55–0.81), VISAGE score (AUC = 0.67, 95%CI 0.55–0.80) and RIS-i score (AUC = 0.69; 95%CI: 0.57–0.80) (Table 4). Then Z-test was used to compare the AUCs, the results showed that the prediction efficiency of this study was higher than that of Godet’s score (*Z* = 2.142, *p* = 0.042) and VISAGE score (*Z* = 1.950, *p* = 0.044), but there was no significant difference compared with RIS-i score (*Z* = 1.184, *p* = 0.343).

**Table 2:** Results of multivariate analysis.

Parameters before extubation	OR	*β*	95%CI	*p*
GCS-M	9.45	2.25	2.59–34.56	0.001
Coughing ability	3.61	1.28	1.08–12.03	0.037
Temperature	2.13	-	0.67–6.74	0.200
Oxygenation index	3.28	1.18	1.03–10.50	0.045

*β* = ln (OR).

**Table 3:** The extubation scoring model of endotracheal neurocritical patients that have passed SBT.

Factors	Points
GCS-M	
≥4	0
<4	2
Coughing ability	
≤II	0
>II	1
Oxygenation index	
>200	0
≤200	1

Coughing ability: I: strong coughing, more than 4 consecutive sounds, and sputum choking out of the catheter; II: medium coughing, 2–4 sounds, and sputum not choked out of the catheter; III: weak coughing, less than 2 sounds; IV: no response to sputum suction.

**Table 4:** Logistic regression analysis results and ROC characteristics of different predictive scoring systems.

	OR	95%CI	*p*	AUC	95%CI	Sensitivity	Specificity	Cutoff value
Thomas Godet’s score	1.32	1.05–1.67	0.006	0.68	0.55–0.81	87	50	>13
VISAGE score	2.21	1.28–3.83	0.010	0.67	0.55–0.80	89.1	37.5	>2
RIS-i score	0.61	0.46–0.80	0.005	0.69	0.57–0.80	84.80	41.70	≤5
Our score	3.37	1.97–5.78	<0.001	0.79	0.68–0.90	79.2	69.6	>1

Based on the observed extubation failure rate, the sample size of 116 cases, and the OR, the statistical power was calculated. For a two-tailed test with *α* = 0.05, the achieved power exceeded 80% for all significant factors, indicating sufficient sensitivity to detect clinically relevant associations.

## 4 Discussion

A large proportion of patients admitted to the neurocritical care unit require endotracheal intubation and mechanical ventilation. Neurocritical care patients are also at high risk of extubation failure, so it is vital to develop reliable clinical tools for guiding decisions on extubation, re-intubation, and tracheostomy. Two recent large-scale studies of factors associated with extubation success and failure (SET and ENIO) have yielded predictive scales for evaluating the safety of extubation (i.e., the risk of extubation failure); however, a recent meta-analysis including more than 17,000 critical stroke patients concluded that the use of the SET score for determining extubation time did not improve neurological outcome (according to the modified Rankin scale [mRS]) or reduce mortality, ICU and hospital stay (LOS), and the duration of mechanical ventilation. Further, the authors suggested that the necessity for tracheostomy should be based on patient characteristics, neurological state, prognosis, risk–benefit ratio, and patient comfort rather than SET score (21). Therefore, we developed a novel predictive model incorporating multiple demographic and clinical factors, including neurological status, and tested its efficacy for distinguishing extubation success from failure among neurocritical care patients. The scale presented here demonstrated superior accuracy for identifying patients ultimately achieving successful intubation compared to Godet’s, VISAGE and RIS-i scales. The scores according to Godet’s, VISAGE, and RIS-i scales did differ significantly between successful and failed extubation groups, and notably all include assessment of consciousness and airway protection, factors identified as independent predictors in the current study. Nonetheless, ROC analysis indicated slightly better performance by the current scale compared to Godet’s, VISAGE, and RIS-i scales.

In VISAGE and Godet’s scales, the level of consciousness is reflected by Coma Recovery Scale-Revised (CRS-R) “visual” subitems, especially visual pursuit. The RIS-i scale also includes items on mental status, bulbar function, oxygenation, and BMI, but mental status is reflected by the RASS, which is usually used to assess the depth of sedation. Therefore, we replaced the RASS with a grade of consciousness (awake 3′, drowsiness 2′, lethargy 1′, coma 0′), and found that RIS-i score was still predictive of successful extubation. Many studies have suggested that a lower total GCS score is predictive of a greater risk for extubation failure. According to univariate logistic regression analysis, however, only the GCS-M has significant predictive value. Indeed, 92.6% of subjects with GCS-M > 4 were successfully extubated (sensitivity), while a large fraction of patient with failed extubation (44%) scored ≤4 on the GCS-M (specificity). Eye opening, as a sub-component of the GCS score, did not have a significant effect on extubation outcomes. Although the total GCS score also demonstrated high sensitivity, its specificity was not ideal and the AUC was lower than that of the GCS-M. It is possible that this lower predictive value resulted from setting the GCS-V as 1 (default). We found that it was more appropriate to use a cognitive item when assessing the level of consciousness, such as the CRS-R “visual pursuit,” or to use a simple grade of consciousness (awake 3′, drowsiness 2′, lethargy 1′, coma 0′). In the present cohort, 1 patient in a minimally consciousness state with the same grade as the patient with drowsiness. There were also 6 patients in unresponsive awake syndrome (vegetative state) with the same grade as those in lethargy.

For decisions on extubation among general ICU patients, arterial blood oxygenation index is considered crucial and is included in both ENIO and RIS-i scales. Samely, this study also confirmed that there was a significant statistical difference in the oxygenation index before extubation between the successful and failed groups, and was finally included in this scoring model. The RIS-i scale also considers patient BMI, and we found that 3 of 24 patients in the extubation failure group (12.5%) had glossoptosis, of which 2 exhibited a BMI > 28 and comorbid obstructive sleep apnea syndrome (OSAS). Therefore, obesity and a history of OSAS may be important predictors of extubation failure, when the number of cases is sufficient, the analysis of these population should be focused.

This study included 116 neurocritical patients who passed the SBT by weaning from ventilators for >2 h prior to tracheal catheter removal. We defined extubation failure as the need for re-intubation within 5 days after extubation rather than within 2 days, thus maximizing the actual extubation failure rate. This study indicates that patients need to be comprehensively evaluated for neurological function, airway protection ability, and physiological and biochemical indexes before extubation. These evaluations include mental status, GCS motor score, swallowing attempt frequency, cough frequency, and body temperature. Based on these main factors, we established a neurocritical extubation risk model including GCS-M, coughing ability, and oxygenation index which could reflect the ability of the airway protection in patients with severe neurological diseases. The model’s successful extubation ability was higher than several other prediction scales by previous studies (VISAGE, Godet’s, and RIS-i score) for neurocritical patients, thus facilitating safer extubation decisions.

This study has several limitations. Due to the small sample size of a single center, many other possible predictive factors, such as sputum properties, BMI, brain lesion size, and primary etiology were not included in this study’s analysis. Secondly, this retrospective design had not done *a priori* sample size estimation, and lack of external validation affected the generalizability and practicability of the conclusions as well. Therefore, we will continue to expand the sample size to test additional factors and validate the existing results in further study.

## 5 Conclusion

We describe a new scoring model consists of 3 factors (GCS-M, coughing ability, and oxygenation index) which can help with decision making of extubation for neurocritical patients. But it still needs further validation.

## Data Availability

The original contributions presented in the study are included in the article/supplementary material, further inquiries can be directed to the corresponding author.
